# Age-related changes in brain structural covariance networks

**DOI:** 10.3389/fnhum.2013.00098

**Published:** 2013-03-26

**Authors:** Xinwei Li, Fang Pu, Yubo Fan, Haijun Niu, Shuyu Li, Deyu Li

**Affiliations:** ^1^State Key Laboratory of Software Development Environment, Beihang UniversityBeijing, China; ^2^Key Laboratory for Biomechanics and Mechanobiology of Ministry of Education, Department of Biomedical Engineering, School of Biological Science and Medical Engineering, Beihang UniversityBeijing, China.

**Keywords:** connectivity, structural covariance network, normal aging, neuroimaging, sensorimotor, neurocognition

## Abstract

Previous neuroimaging studies have suggested that cerebral changes over normal aging are not simply characterized by regional alterations, but rather by the reorganization of cortical connectivity patterns. The investigation of structural covariance networks (SCNs) using voxel-based morphometry is an advanced approach to examining the pattern of covariance in gray matter (GM) volumes among different regions of the human cortex. To date, how the organization of critical SCNs change during normal aging remains largely unknown. In this study, we used an SCN mapping approach to investigate eight large-scale networks in 240 healthy participants aged 18–89 years. These participants were subdivided into young (18–23 years), middle aged (30–58 years), and older (61–89 years) subjects. Eight seed regions were chosen from widely reported functional intrinsic connectivity networks. The voxels showing significant positive associations with these seed regions were used to describe the topological organization of an SCN. All of these networks exhibited non-linear patterns in their spatial extent that were associated with normal aging. These networks, except the primary motor network, had a distributed topology in young participants, a sharply localized topology in middle aged participants, and were relatively stable in older participants. The structural covariance derived using the primary motor cortex was limited to the ipsilateral motor regions in the young and older participants, but included contralateral homologous regions in the middle aged participants. In addition, there were significant between-group differences in the structural networks associated with language-related speech and semantics processing, executive control, and the default-mode network (DMN). Taken together, the results of this study demonstrate age-related changes in the topological organization of SCNs, and provide insights into normal aging of the human brain.

## Introduction

The majority of neuroimaging studies of aging have reported a consistent pattern of gray matter (GM) volumetric reductions in the human cortex, involving mainly prefrontal regions, parietal, and temporal association cortices, and the insula and cingulum (Resnick et al., 2003; Sowell et al., 2003; Raz et al., 2005; Du et al., 2006; Terribilli et al., 2011). However, more and more studies have demonstrated that cerebral changes with normal aging are not simply characterized by regional alterations but rather by the reorganization of cortical connectivity patterns (O'Sullivan et al., 2001; Koch et al., 2010; Wu et al., 2011b; Zhu et al., 2012). Using diffusion tensor imaging (DTI), several studies have consistently reported a diffuse loss of axonal integrity in senior populations (Salat et al., 2005; Pagani et al., 2008; Madden et al., 2009), which allowed for inferences regarding changes of structural connectivity in older people compared with younger adults. Building on complex network analysis methods, Gong et al. (2009) reported a reduction in overall cortical connectivity, decreased local efficiency, and a shift in regional efficiency from parietal and occipital to frontal and temporal neocortex in older brains.

The investigation of structural covariance networks (SCNs) using structural magnetic resonance imaging (sMRI) is another useful method to explore structural brain networks. This approach mainly characterizes the pattern of structural covariance in GM morphology (e.g., volume, thickness and surface area) between brain regions using a general linear model (GLM) framework (Mechelli et al., 2005; Lerch et al., 2006; Nosarti et al., 2010; Zielinski et al., 2010; Montembeault et al., 2012; Soriano-Mas et al., 2012). Many studies have demonstrated underlying relationships among brain areas using structural correlation by sMRI, anatomical connectivity by DTI, and functional correlation by resting-state functional MRI. For example, He and colleagues found that structural networks based on cortical thickness measurements were compatible with known functional networks (He et al., 2007). Greicius et al. measured functional connectivity using independent component analysis and anatomical connectivity using DTI and found there existed white matter tract structural connections between functionally connected regions (Greicius et al., 2009). A recent study reported agreement in the correlations in GM thickness and underlying fiber connections across brain areas, but more information was included for the thickness network than the fiber network (Gong et al., 2012). In addition, Seeley and colleagues demonstrated that SCNs using voxel-based morphometry (VBM) were able to recapitulate the functional connectivity network topologies (Seeley et al., 2009). The covariance of different cortical regions in their GM volumes was considered to be the result of mutual trophic influences (Ferrer et al., 1995) or common experience-related plasticity (Draganski et al., 2004; Mechelli et al., 2004). The consistency among these three networks provides substantial support for SCNs serving a measure of network integrity in cross-sectional studies.

Of note, the analysis of SCNs using VBM has been successfully applied to map the eight SCNs and explore how neural systems build large-scale structural covariance during development (Zielinski et al., 2010). One previous aging study using structural covariance approaches compared two populations (younger vs. older subjects) and reported reduced structural associations in order adults, specifically in high-order cognitive networks (Montembeault et al., 2012). However, because subjects in only two age categories were involved in that experiment, it could not determine the aging trajectories of these sensorimotor and high-order cognitive networks. Zielinski et al. (2010) found that there were non-linear trajectories of primary visual, auditory, and sensorimotor networks during development. Moreover, these networks were provisionally established by early childhood, but underwent significant expansion in early adolescence before contraction or pruning in late adolescence. Many studies have shown that age-related atrophy of some neural regions follows variable, non-linear patterns (Allen et al., 2005; Kennedy et al., 2009; Terribilli et al., 2011). However, it remains unknown whether and how age-related changes of primary sensorimotor and high-order cognitive SCNs exhibit non-linear trajectories during normal aging.

Here, we used an SCN mapping approach to investigate eight large-scale networks in 240 healthy participants aged 18–89. These participants were subdivided into young (18–23 years), middle aged (30–58 years), and older (61–89 years) subjects. Given that SCNs subserving language, social–emotional, and executive control functions have shown gradual deterioration through normal aging, we expected to observe age-related trajectories in the deterioration of these large-scale networks. To test our hypothesis, we first investigated the aging trajectories of three sensorimotor and five high-order cognitive SCNs in three groups composed of an equal number of subjects at different ages, and then compared these SCNs differences between the groups.

## Materials and methods

### Participants

Three hundred sixteen right-handed, healthy subjects were selected from the Open Access Series of Imaging Studies (OASIS) cross-sectional database (http://www.oasis-brains.org) (Marcus et al., 2007). Data from seven subjects were excluded from further analysis because of poor image quality or image preprocessing. Because of relatively few subjects aged 30–60 years, we first selected 80 subjects from this age range as the middle-aged group (30 males and 50 females). Then, we selected 80 separate subjects around 75 years of age as the old group. Finally, we selected the youngest 80 subjects matched for gender as the young group. The names and characteristics of the groups are shown in Table 1. All subjects were evaluated using the Mini-Mental State Examination (MMSE) (Folstein et al., 1975) and Clinical Dementia Rating (CDR) scales (Morris, 1993; Morris et al., 2001). MMSE scores were higher than 29, and CDR scores were all zero. For demographic data on all subjects, see Marcus et al. (2007). This dataset has been used in several previous studies (Bakkour et al., 2009; Fjell et al., 2009; Salat et al., 2009; Li et al., 2011).

**Table 1:** **Characteristics of the subjects in this study**.

**Group ID**	**Age (mean ± SD)**	**Number of subjects**
Young (Y)	18–23 (20.66 ± 1.47)	80 (F:50/M:30)
Middle-aged (M)	30–58 (47.43 ± 8.23)	80 (F:50/M:30)
Old (O)	61–89 (73.75 ± 7.12)	80 (F:55/M:25)

Abbreviations *F* *female* *M* *male*.

### Image acquisition

For each subject, three to four individual T1-weighted magnetization-prepared rapid gradient echo (MP-RAGE) images were acquired on a 1.5T Vision scanner (Siemens, Erlangen, Germany) within a single session. Head movement was minimized with cushioning and a thermoplastic facemask. Images were motion corrected and averaged to create a single image with a high contrast-to-noise ratio. MP-RAGE parameters were empirically optimized for gray/white contrast: *TR* = 9.7 ms; *TE* = 4 ms; flip angle = 10°; slice number = 128; resolution = 256 × 256 (1 × 1 mm); thickness = 1.25 mm.

### Image preprocessing

Structural MR images were processed using a technical computing software program (MATLAB 2010; The MathWorks Inc., Natick, Mass) and Statistical Parametric Mapping software (SPM 8; The Wellcome Department of Imaging Neuroscience, London, UK). Following the inspection of image artifacts, image preprocessing was performed with the VBM8 toolbox (http://dbm.neuro.uni-jena.de/vbm/). Briefly, all native-space MRIs were segmented to extract GM based on an adaptive maximum *a posteriori* technique (Rajapakse et al., 1997) and partial volume estimation method (Tohka et al., 2004) without the need for *a priori* tissue probability information. In addition, a spatially adaptive non-local denoising filter (Manjon et al., 2010) and a hidden Markov random field model (Rajapakse et al., 1997) were applied to minimize the level of noise in the resulting GM segments. Subsequently, the high-dimensional DARTEL (diffeomorphic anatomical registration using exponentiated lie algebra) approach provided non-linear deformation to normalize the images to the DARTEL template in Montreal Neurological Institute (MNI) space, which was derived from 550 healthy control subjects (Ashburner, 2007). Non-brain tissue was also removed. Additionally, the Jacobian determinants derived from the spatial normalization were used to modulate the GM value for each voxel to preserve the total amount of GM from the original images (Good et al., 2001). We used non-linear components only, which allowed us to analyze relative (i.e., corrected for individual brain size) differences in regional GM volume. Finally, the resulting modulated and normalized images were smoothed with a 12 mm full width at half maximum isotropic Gaussian kernel.

### Extraction of seed volumes

**Figure A1:**
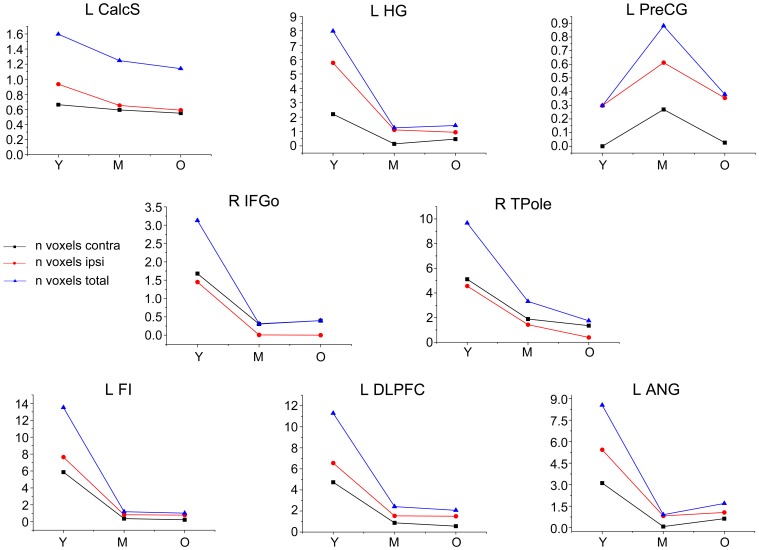
**Trajectories of the structural covariance networks derived from contralateral seeds.** The plots of voxel counts by group (*P* < 0.05, FWE) indicate a similar pattern to the ipsilateral seed, except for the auditory and speech networks, which show differences in their spatial extents. The y-axis represents the voxel number (×10^4^). AbbreviationsCalcscalcarine sulcusHGHeschl's gyrusPreCGprecentral gyrusIFGoinferior frontal gyrus, pars opercularisTPoletemporal poleANGangular gyrusDLPFCdorsolateral prefrontal cortexFIfrontoinsular cortexRrightLleftYyoung groupMmiddle-aged groupOold group.

To assess the structural covariance pattern of each large-scale network, we extracted individual GM volumes from eight seed regions of interest (ROIs). These ROIs included the primary visual, auditory, and motor cortex, as well as language-related speech and semantic areas, areas related to salience and executive control, and the default-mode network (DMN). We based our ROIs on previous studies (Zielinski et al., 2010; Montembeault et al., 2012). For each region, seeds were defined with the MarsBar ROI toolbox (http://marsbar.sourceforge.net/) as 4-mm radial spheres centered at the following MNI coordinates: right calcarine sulcus (9, −81, 7), right Heschl's gyrus (46, −18, 10), right precentral gyrus (28, −16, 66), left inferior frontal gyrus, pars opercularis (IFGo) (−50, 18, 7), left temporal pole (−38 10, −28), right frontoinsular cortex (38, 26, −10), right dorsolateral prefrontal cortex (DLPFC) (44, 36, 20), and right angular gyrus (46, −59, 23). In addition, we selected contralateral seeds by changing the sign on each seed's x coordinate to perform similar SCNs analyses (Table A1, and Figure A1).

### Statistical analysis

A voxel-wise statistical analysis was performed on the GM images using the GLM as applied in SPM8. Eight multiple regression models were used to test the strength of the structural covariance between each seeds and all other regions across whole-brain GM for each age group. In each regression model, the extracted mean GM volume from each ROI was entered as a covariate of interest, and gender as a confounding covariate. Because of the unequal sample size across the genders, we removed the effects of gender on the SCN patterns in the correlation analysis. These statistical analyses identified voxels that had a positive covariance with each *a priori* selected ROI in each group. The resulting correlation maps were using height and extent thresholds at *P* < 0.05 with family-wise error (FWE) correction. They were displayed on the MNI template to allow qualitative comparisons between the age groups using BrainNet Viewer software (http://www.nitrc.org/projects/bnv/). To quantify differences in normal aging trajectories across networks, we calculated the total number of significant positive ipsilateral, contralateral, and whole-brain voxels and plotted these across the age groups.

To further examine the effects of age on specific regional covariance, we performed between-group difference analyses of SCN patterns for any two groups according to the scientific literature (Lerch et al., 2006). For any pair of voxels in two groups, their structural correlation may have different slopes, and the difference in slope may represent the difference in their structural association. The difference in slope was tested using a classic interaction linear model:

$$V_{i}=\beta_{0}+\beta_{1}V_{j}+\beta_{2}\text{Group}+\beta_{3}(V_{j}\times \text{Group})+\varepsilon$$

where *V*_*i*_ and *V*_*j*_ represented the GM volumes of a pair of voxels in two groups. The Group component was modeled using treatment contrasts, and significance tested using the Student's *t* statistic. Specific *t*-value contrasts were established to map the voxels that expressed a significantly different structural association between any two groups. The threshold for the resulting statistical parametric maps was given at *P* < 0.05 for the height and extent thresholds, with FWE multiple comparisons correction.

## Results

For each network, the age-related SCN trajectory was identified by the spatial extent of each SCN. These results were demonstrated by functional domain, as described in the following sections. We also explored regions that significantly differed in their structural associations between any two groups.

### Primary sensory and motor networks

**Figure 1:**
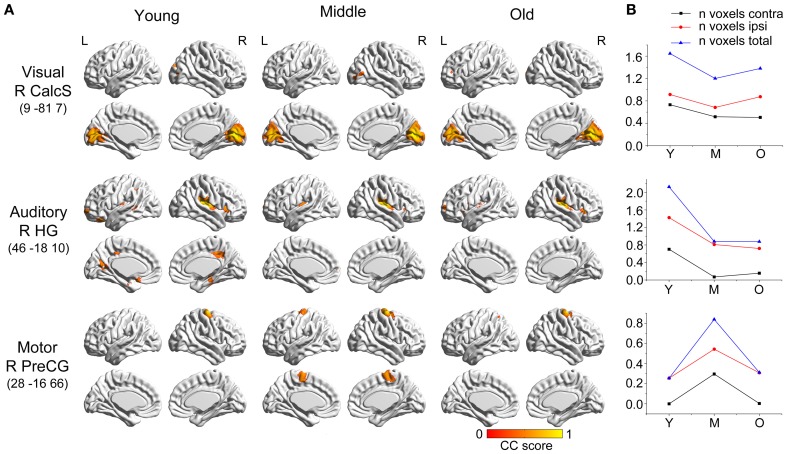
**Age-related changes in primary sensory and motor structural covariance networks. (A)** Statistical maps of regions significantly correlated with the seed region in each group. The results are presented as correlation coefficient values (*P* < 0.05, FWE corrected). **(B)** The plots of voxel counts by group indicate small covariance changes throughout the life-span. The y-axis represents the voxel number (×10^4^). AbbreviationsCalcscalcarine sulcusHGHeschl's gyrusPreCGprecentral gyrusRrightLleftYyoung groupMmiddle-aged groupOold groupCCcorrelation coefficient.

Seeds within primary visual cortex (right calcarine sulcus) produced SCNs with a relatively preserved pattern (Figure 1). However, there were small changes in the distributions of the covariance maps. In the young and middle-aged groups, the covariance regions mainly included the bilateral calcarine sulcus, lingual gyrus, cuneus and right lateral occipital gyrus, whereas only included bilateral calcarine sulcus, lingual gyrus, and cuneus in the old group. Primary auditory cortex (i.e., the right Heschl's gyrus) covaried with the bilateral insula and precuneus, the right posterior cingulate cortex (PCC), parahippocampus, and inferior frontal gyrus, and the left orbital-frontal cortex in the young participants, and underwent significant contraction in the middle-aged and old groups to include only the bilateral insula regions. There was a flat transition in the covariance maps during aging. Primary motor cortex (right precentral gyrus) correlated with the ipsilateral precentral regions in the young participants, which progressed at middle age to include the contralateral precentral and supplementary motor areas. In old participants, these structural associations were similar to the young participants.

In addition, within the primary sensory and motor networks, there were no significant differences observed in the structural association between any two groups when we compared the slopes of the structural associations on a voxel-by-voxel basis.

### Language-related speech and semantic networks

**Figure 2:**
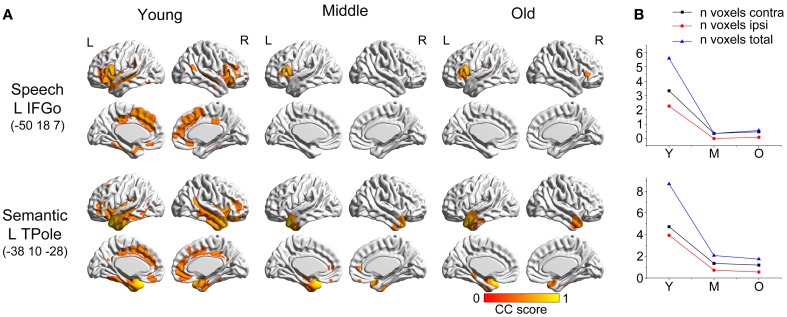
**Age-related changes in speech and semantic structural covariance networks. (A)** Statistical maps of regions significantly correlated with the seed region in each group. The results are presented as correlation coefficient values (*P* < 0.05, FWE corrected). **(B)** The plots of voxel counts by group indicate abrupt contraction in the middle-aged group and mild changes in the old group. The y-axis represents the voxel number (x× 10^4^). AbbreviationsIFGoinferior frontal gyrus, pars opercularisTPoletemporal poleLleftRrightYyoung groupMmiddle-aged groupOold groupCCcorrelation coefficient.

The two language-related speech and semantic networks followed similar variation patterns (Figure 2). From the young participants to the middle group, the covariance regions showed abrupt decreases. They then showed smaller changes at the following ages. For the language-related speech network, the covariance map involved the bilateral anterior insula, medial frontal, cingulate cortex, and temporal regions in the young participants, and then contracted to include seed autocorrelation in the older participants. The semantic regions correlated with the bilateral temporal cortices, cingulate gyrus, insula and frontal regions in the young participants, and shrank sharply to include only the anterior temporal cortices during normal aging.

**Figure 3:**
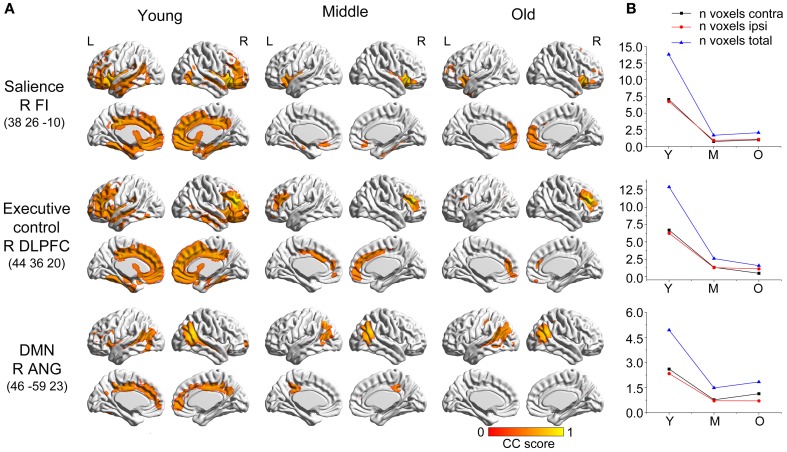
**Age-related changes in salience, executive control, and default-mode structural covariance networks. (A)** Statistical maps of regions significantly correlated with the seed region in each group. The results are presented as correlation coefficient values (*P* < 0.05, FWE corrected). **(B)** The plots of voxel counts by group indicate abrupt contraction in the middle-aged group and mild changes in the old group. The y-axis represents the voxel number (× 10^4^). AbbreviationsDMNdefault-mode networkDLPFCdorsolateral prefrontal cortexFIfrontoinsular cortexANGangular gyrusRrightLleftYyoung groupMmiddle-aged groupOold groupCCcorrelation coefficient.

**Figure 4:**
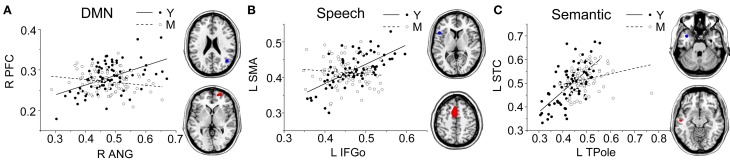
**Group differences for the young vs. the middle-aged subjects.** Significant between-group differences within the DMN **(A)**, speech **(B)**, and semantic **(C)** structural covariance networks were found. For each network, the region of interest (upper) and region showing the most significant structural association (lower) are presented on the right, and a plot of slop differences between the seed region and a 4-mm radius sphere centered on the peak voxel are presented in the left. Voxels with *P*_FWE_ < 0.05 are displayed. AbbreviationsDMNdefault-mode networkANGangular gyrusIFGoinferior frontal gyrus, pars opercularisTPoletemporal polePFCprefrontal cortexSMAsupplementary motor areaSTCsuperior temporal cortexRrightLleftYyoung groupMmiddle-aged group.

**Figure 5:**
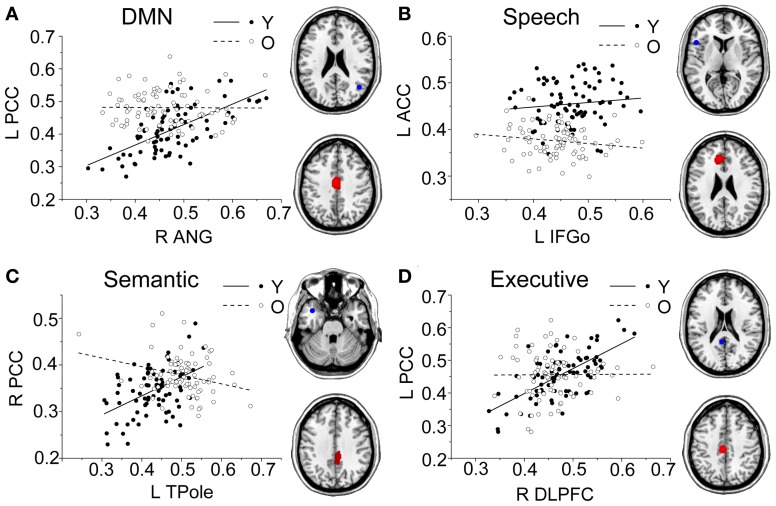
**Group differences for the young vs. the old subjects.** Significant between-group differences within the DMN **(A)**, speech **(B)**, semantic **(C)**, and executive **(D)** structural covariance networks were found. For each network, the region of interest (upper) and region showing the most significant structural association (lower) are presented on the right, and a plot of slop differences between the seed region and a 4-mm radius sphere centered on the peak voxel are presented in the left. Voxels with *P*_FWE_ < 0.05 are displayed. AbbreviationsDMNdefault-mode networkANGangular gyrusIFGoinferior frontal gyrus, pars opercularisTPoletemporal poleDLPFCdorsolateral prefrontal cortexPCCposterior cingulate cortexACCanterior cingulate cortexRrightLleftYyoung groupOold group.

Comparing the other SCNs, language-related speech and semantic networks showed more age-related changes. Specifically, the left supplementary motor and superior temporal areas showed significant positive associations with the speech seed region in young adults, whereas this covariance disappeared in the middle-aged group (Table 2 and Figure 3). Decreased positive associations between the left superior temporal region and left temporal pole were found in the middle-aged group compared with the young group (Table 2 and Figure 4). There were significant positive associations between the left anterior and the PCC and left IFG in the young participants, and this covariance became a negative correlation in the old group (Table 2 and Figure 4). When compared with the old group, there was a significant positive association between the right PCC and the semantic seed region in the young participants (Table 2 and Figure 5).

**Table 2:** **Contrast analysis of structural covariance network trajectories**.

**Network**	**Contrast**	***x***	***y***	***z***	**Region**	**BA**	**Voxel size**	**maxT**
DMN (R ANG)	Y > M	27	65	1	R PFC	10	181	4.97
	Y > O	−3	−10	40	L PCC	23/24/31	1100	5.34
		−6	33	25	L ACC	32	110	4.43
Speech (L IFGo)	Y > M	−3	12	52	L SMA	6/8	3498	5.35
		−51	−16	−11	L STC	21/22	741	4.77
		54	−28	3	R STC	21/22	630	4.52
	Y > O	−10	35	25	L ACC	8/9/32	3235	5.82
		−5	29	52	L SFC			5.28
		−4	−21	37	L PCC	24/31	218	4.29
Semantic (L TPole)	Y > M	−54	−15	−11	L STC	22	114	4.31
	Y > O	10	−34	36	R PCC	23/31	978	5.33
		−40	−3	10	L Insula	13	217	4.72
		26	33	−21	R OFC	11	200	4.33
Executive (R DLPFC)	Y > O	−4	−22	43	L PCC	24/31	419	4.99

*The regions listed showed significant between-group differences (P < 0.05, FWE corrected). x, y, z coordinates are reported in standard MNI space*.

Abbreviations *DMN* *default-mode network* *ANG* *angular gyrus* *IFGo* *inferior frontal gyrus, pars opercularis* *TPole* *temporal pole* *DLPFC* *dorsolateral prefrontal cortex* *PFC* *prefrontal cortex* *PCC* *posterior cingulate cortex* *ACC* *anterior cingulate cortex* *SMA* *supplementary motor area* *STC* *superior temporal cortex* *SFC* *superior frontal cortex* *OFC* *orbitofrontal cortex* *R* *right* *L* *left* *Y* *young group* *M* *middle-aged group* *O* *old group* *BA* *Brodmann area.*

### Salience, executive control, and default-mode networks

The three networks associated with social–emotional and cognitive function showed similar patterns to the language-related speech and semantic networks, with more distributed structural covariance in the young participants and limited covariance patterns throughout the later stages (Figure 5). Specifically, the right frontoinsular cortex anchored covariance maps included extensive areas of the bilateral lateral and medial frontal cortex, temporal cortices, and cingulate regions. This SCN underwent significant shrinkage in the middle-aged group to include only bilateral insular regions. In the older subjects, this network somewhat extended to include the bilateral medial prefrontal regions. The right DLPFC seed covaried with the bilateral frontal, temporal, and cingulate cortices in the young participants, but shrank into a more focal distribution in the middle-aged and older subjects. A seed in right angular gyrus produced an aging SCN, including the bilateral angular gyrus, middle temporal, cingulum, and prefrontal regions in the young participants, and contracting to include the bilateral PCC and angular gyrus in the middle-aged group, and only the bilateral angular gyrus in the old group.

There were no significant differences observed in the structural associations between any two groups within the salience network. The left PCC showed a significant positive association with the executive control seed region and right angular region (DMN seed) in the young adults, whereas this covariance disappeared in the old group (Table 2 and Figure 5). Furthermore, a less robust positive association between the right prefrontal region and DMN seed region was found in the middle-aged group compared with the young participants (Table 2 and Figure 4).

## Discussion

In this study, we investigated the age-related trajectories of eight large-scale networks using an SCN mapping approach in three distinct age groups. All networks exhibited non-linear patterns across normal aging in terms of the spatial extent of the network. Except for the primary motor network, these networks showed a more distributed topology in young participants, which shrank sharply to a more localized topology in the middle-aged group, and maintained this localized topology in the old group. Primary sensory and motor networks showed fewer age-related changes compared with high-order cognitive networks. Moreover, there were significant between-group differences in language-related speech and semantic networks, the executive control network, and the DMN. Taken together, our results provide evidence of variations in the topological organization of SCNs during normal aging.

### Comparison of structural covariance networks and other networks

The cerebral cortex is organized into networks of functionally complementary areas. New advances in modern neuroimaging techniques and quantitative analysis of complex networks have made the investigation of brain network topological organization possible. DTI is a useful tool for non-invasively mapping cortico-cortical anatomical connections by examining axonal integrity. Insight into the structural covariance of GM morphology (e.g., volume, thickness, and surface area) between brain regions is provided by sMRI. Resting-state fMRI has allowed for assessments of the strength of functional connections within a network by quantifying correlated activity [i.e., spontaneous, low-frequency fluctuations in the blood oxygen level-dependent (BOLD) signal] between brain regions at rest.

The current studies have demonstrated the underlying relationships among structural correlations, anatomical connectivity, and functional correlations. For example, structural networks based on cortical thickness measurements were consistent with known functional networks (He et al., 2007). A recent study reported agreement in the correlations in GM thickness and underlying fiber connections across brain areas, but more information was included for the thickness correlation network than the fiber connection network (Gong et al., 2012). In addition, SCNs determine using VBM recapitulated the canonical intrinsic connectivity networks topologies (Seeley et al., 2009). The consistency among the SCNs and other networks provides substantial support for the use of SCNs as a measure of network integrity for cross-sectional studies.

### Age-related changing trajectory of SCNs

**Figure A2:**
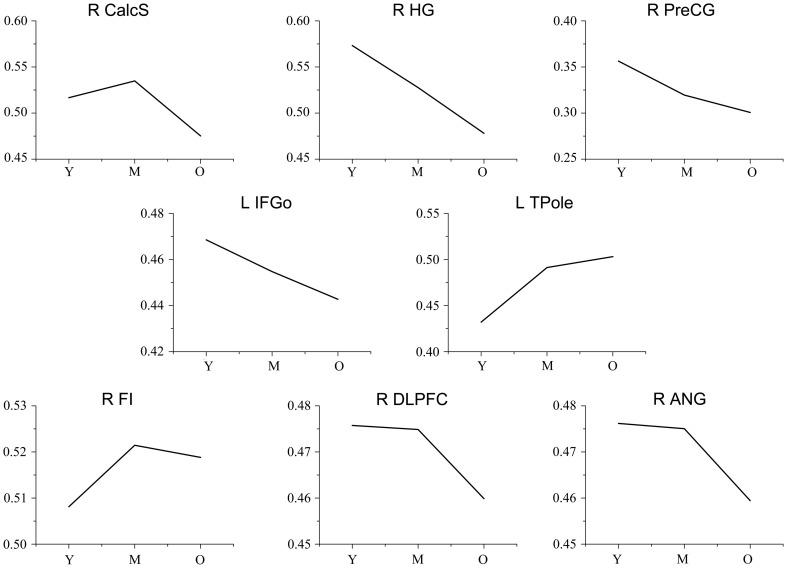
**Trajectories of the gray matter volume for each seed.** The y-axis represents the mean gray matter volume for each age group. AbbreviationsCalcscalcarine sulcusHGHeschl's gyrusPreCGprecentral gyrusIFGoinferior frontal gyrus, pars opercularisTPoletemporal poleANGangular gyrusDLPFCdorsolateral prefrontal cortexFIfrontoinsular cortexRrightLleftYyoung groupMmiddle-aged groupOold group.

In this study, we employed three age groups to map the trajectory of SCN changes over age. Except the primary motor network, these SCNs appeared the similar non-linear pattern that had a distributed topology in young participants, a sharply localized topology in middle aged participants, and were relatively stable in older participants. This trajectory was similar to age-related changes of integrated local efficiency of brain network (Wu et al., 2011b). They employed the graph theory analysis method and reported that the local efficiency in the young group was significantly larger than those of the middle and old groups, whereas no significant difference was found between the middle and old groups. The shrinkage of SCNs as well as the reduction of local efficiency might be explained by the regionally distributed pattern of GM atrophy (Bergfield et al., 2010). As for primary motor network, we found inverted V-curve tendency among three age groups in the SCN of primary motor cortex. The previous study reported increased inter-regional correlations between bilateral primary motor cortex in the aging brain (Chen et al., 2011). In our study, we found the structural covariance of primary motor cortex between two hemispheres in the middle group. Alternatively, we found that the volume of right precentral gyrus reduced with age increasing (Figure A2), which might reflect a compensation mechanism that a network may need to work harder by becoming overactive due to regional volumetric atrophy with the network (Reuter-Lorenz and Cappell, 2008).

### Intragroup patterns and between-group differences of SCNs

#### Primary sensory and motor networks

In this study, we found that seeds within the primary visual, auditory, and motor cortices produced SCNs with smaller spatial extents (i.e., total voxel count exhibiting a significant correlation at the corrected threshold) compared with other networks. This is consistent with previous work (Lerch et al., 2006) with mapped anatomical correlations across cerebral cortex (MACACC) using cortical thickness. Similarly, they used the number of cortical region showing significant correlations with seed region to describe the strength of MACACC. They found the primary motor, sensorimotor, and visual areas had the lowest strengths of correlation, whereas the association cortices had the highest strength. This could be explained by the functions of association cortices, because these cortices receive and integrate inputs from multiple cortical and non-cortical sources, and distribute information to multiple areas.

In addition, primary sensory and motor networks showed fewer age-related changes compared with high-order cognitive networks. There were no significant differences observed in the structural association between any two groups when the slopes of the structural associations were contrasted on a voxel-by-voxel basis within the primary sensory and motor networks. This finding is in line with a recent study (Montembeault et al., 2012) in which between-group differences were only observed in high-order cognitive SCNs between young and old subjects, rather than primary sensory and motor networks.

#### Language-related speech and semantic networks

The language-related speech and semantic networks showed significant age-related changes, with a more localized topology with increasing age. These results could account for the decline of language abilities across in normal aging. Federmeier and colleagues observed age-related changes in the timing with which message-level information impacted semantic analysis using event-related potentials, which indicated that aging affects high-order language processes (Federmeier et al., 2003). Wierenga and colleagues also reported that healthy older adults had increased difficulty in word retrieval with unchanged semantic knowledge (Wierenga et al., 2008).

Within the language-related speech SCN, a reduced structural association was observed the between inferior frontal gyrus and the supplementary motor area in the middle-aged group compared with the young participants. The speech SCN linked the language and motor systems that enable speech fluency (Seeley et al., 2009). This reduced structural covariance may explain the decline of motor speech skills with the transference of language to a speech code in older adults. Similarly, there was a significant decrease in the covariance between the seed region and the anterior cingulate cortex (ACC) in the old group compared with the young participants. This could be related to the functional degradation of these areas. Takahashi et al. reported that significant age-related reductions in regional cerebral blood flow in the left IFG and the bilateral medial frontal gyri and ACC using single-photon emission tomography (Takahashi et al., 2005). In addition, as the ACC plays a critical role in the control of speech responses (Paus et al., 1993), this reduced covariance may explain some speech difficulties in senior populations.

In semantic SCN analysis, a reduced structural association between the left temporal pole and left superior temporal cortex was found in the middle-aged group compared with the young participants. Many studies have shown that the superior temporal cortex is associated with understanding spoken words (Demonet et al., 1992; Chao et al., 2002; Okada and Hickok, 2006). Thus, this reduced structural association may be related to a decline in auditory word comprehension with increasing age. In the comparisons of the young and old participants, the main differences we found were in the structural associations between the left temporal pole and PCC. The results of functional neuroimaging studies in young, healthy adults provide compelling evidence for the involvement of the PCC in memory retrieval. For example, PCC activation was elicited during recognition of thematic narrative information learned during training sessions (Maguire et al., 1999). The reduced structural association between these two regions may explain the decline of semantic memory in senior populations.

#### Salience, executive control, and default-mode networks

The three high-order cognitive SCNs showed a similar pattern to the language SCNs, showing a distributed topology in young participants that shrank sharply to a localized topology in the middle-aged group, and maintained a localized topology in the old group. Cepeda and colleagues found age-related decline of executive control processes by examining task-switching performance (Cepeda et al., 2001). Kelly and colleagues also demonstrated the stability of executive control decreased in older subjects (West et al., 2002). Several studies have shown that the DMN is altered in old subjects (Andrews-Hanna et al., 2007; Koch et al., 2010; Sambataro et al., 2010), especially in anterior regions (Damoiseaux et al., 2008). Similarly, Chen et al. found the decreased inter-regional correlations with the DMN module (Chen et al., 2011). Cognitive decline with age, such as cognitive control (Persson et al., 2007) and working memory (Sambataro et al., 2010), is associated with decreased DMN connectivity.

In the executive-control SCN analysis, the main differences we found were in the structural association between the right DLPFC and PCC, which exhibited a positive correlation in the young participants and no association in the old group. The DLPFC shows increased activity when experimental stimuli are presented, and is thought to support on-task processing in attention tasks. The PCC, in contrast, shows decreased activity during stimulus presentation, and is thought to support off-task processing in attention tasks. Some studies have reported that activity in the DLPFC shows a negative correlation with activity in the PCC during the resting state (Greicius et al., 2003; Fransson, 2005), and the negative coupling between these regions is lower in older adults (Sambataro et al., 2010). Our findings are inconsistent with functional connectivity between these areas, which should be further investigated in future studies.

Finally, for the DMN, we found a reduction in the structural association between the right angular gyrus and right prefrontal cortex from the young to the middle-aged participants. In the comparison of the young and old group, we found age-related differences in the structural association between the right angular gyrus and PCC. Similarly, Wu and colleagues reported that the left angular gyrus has a significantly reduced correlation with the PCC in older compared with younger participants in a resting-state fMRI study (Wu et al., 2011a). As the PCC is a prominent region within the DMN, the reduced structural association between the seed regions and the PCC indicates that the DMN SCN shrinks with age.

### Laterality of the SCNs

We found similar SCNs across both hemispheres (Figure A1), consistent with previous SCN analyses of the developing brain (Zielinski et al., 2010). However, the auditory and speech networks showed a notable exception. The SCN derived from the left seed of Heschl's gyrus was distinctly smaller than the one derived from the right seed. In contrast, the SCN derived from the left seed of the IFGo was distinctly larger than the one derived from the right seed, which may explain why speech networks are left-dominated (Powell et al., 2006; Xiang et al., 2010).

### Further considerations

To build upon this study, several issues need to be addressed. First, we assigned subjects in their 30s to the middle-aged group because there were fewer subjects in their 40s and 50s in our sample. This resulted in wide age range in the middle-aged group. In future, this age range should be refined by including more subjects. Second, a previous study has reported the brain functional connectivity pattern could be affected by the sex factor (Biswal et al., 2010). In future, it is interesting to explore the sexual differences when the SCNs change with age. Third, it should be highlighted that this study used ROIs based on previous studies (Zielinski et al., 2010; Montembeault et al., 2012). Future studies using ROIs extracted from ICNs of the same subjects may obtain more accurate definitions of seed regions.

## Conclusions

In this study, we used an SCN mapping approach to investigate eight large-scale networks in a large cohort of healthy participants. Our results show age-related changes in the topological organization of SCNs and provide insights into the normal aging process of the human brain.

### Conflict of interest statement

The authors declare that the research was conducted in the absence of any commercial or financial relationships that could be construed as a potential conflict of interest.

## Appendix

**Table A1:** **Contrast analysis of structural covariance network trajectories derived from contralateral seeds**.

**Network**	**Contrast**	***x***	***y***	***z***	**Region**	**BA**	**Voxel size**	**maxT**
DMN (R ANG)	Y > O	36	24	−20	R IFC	47	1149	5.56
		−36	15	−23	L TPole	38/47	808	5.15
		−44	42	−3	L IFC	47	128	4.53
		4	−27	42	R PCC	24/31	139	4.5
Speech (L IFGo)	Y > M	−50	−55	22	L ANG	39	57	4.23
	Y > O	4	−54	28	R PCC	7/31	2443	5.4
Semantic (L TPole)	Y > M	62	−21	−23	R ITC	20	200	4.38
	Y > O	28	35	−6	R IFC	11/47	361	4.93
		−30	−36	−15	L FG	37	138	4.41
Salience (R FI)	Y > O	5	−47	36	R PCC	23/31/32	1575	5.64
		−39	−3	7	L Insula	13	166	4.65
		−26	−35	−22	L FG	37	392	4.67
Auditory (R HG)	Y > M	26	44	−24	R OFC	11	906	5.30
	Y > O	−8	21	34	L ACC	24/32	864	4.86

*The regions listed showed significant between-group differences (P < 0.05, FWE corrected). x, y, z coordinates are reported in standard MNI space*.

Abbreviations *DMN* *default-mode network* *ANG* *angular gyrus* *IFGo* *inferior frontal gyrus, pars opercularis* *TPole* *temporal pole* *FI* *frontoinsular cortex* *HG* *Heschl's gyrus* *IFC* *inferior frontal cortex* *PCC* *posterior cingulate cortex* *ITC* *inferior temporal cortex* *FG* *fusiform gyrus* *OFC* *orbitofrontal cortex* *ACC* *anterior cingulate cortex* *R* *right* *L* *left* *Y* *young group* *M* *middle-aged group* *O* *old group* *BA* *Brodmann area.*
